# The Use of Fibrinolytic Agents in the Salvage of Free Flaps: A Systematic Review

**DOI:** 10.3390/jpm14080800

**Published:** 2024-07-29

**Authors:** Patrick Mandal, Maximilian Moshammer, Andrzej Hecker, Christian Smolle, Martina Carnieletto, Marcel Mayrhofer, Michael Schintler, Raimund Winter, Lars Peter Kamolz

**Affiliations:** 1 Division of Plastic, Aesthetic and Reconstructive Surgery, Department of Surgery, Medical University of Graz, Auenbruggerplatz 34/4, 8010 Graz, Austria; maximilian.moshammer@joanneum.at (M.M.); christian.smolle@medunigraz.at (C.S.); martina.carnieletto@medunigraz.at (M.C.); marcel.mayrhofer@medunigraz.at (M.M.); michael.schintler@medunigraz.at (M.S.); r.winter@medunigraz.at (R.W.); lars.kamolz@medunigraz.at (L.P.K.); 2 COREMED—Centre for Regenerative Medicine and Precisions Medicine, Neue Stiftingtalstrasse 2, 8010 Graz, Austria

**Keywords:** fibrinolytic agents, free-flap, salvage, microvascular compromise

## Abstract

Background: Microvascular thrombosis following free tissue transfer presents a complex challenge for surgeons and carries the potential risk of flap failure. The application of fibrinolytic agents represents a robust therapeutic option. The aim of this systematic review is to provide a comprehensive overview of the clinical use of fibrinolytic drugs in the rescue of compromised free flaps. Methods: A systematic literature search for clinical studies detailing the utilization of fibrinolytic agents for salvaging free flaps was conducted using the PubMed and Web of Science databases. The inclusion criteria encompassed English-language publications that specifically addressed the clinical application of fibrinolytic agents for free-flap salvage. Results: A total of 331 articles were screened after excluding duplicates, with 56 meeting the inclusion criteria. Among these, 21 were clinical trials (evidence level III), and 35 were case studies (evidence level IV/V). In total, 459 flaps underwent treatment with fibrinolytic agents. Conclusion: The application of fibrinolytic agents appears to be a valuable intervention for rescuing compromised free flaps attributable to microvascular compromise. Notably, no prospective randomized trials have been published on this subject, and the evidence within the existing literature is characterized by its limited and heterogeneous nature. Further research is imperative to gather data on the efficacy, dosage, and safety profile of fibrinolytic agents.

## 1. Introduction

The introduction of free-flap surgery brought a paradigm shift in reconstructive surgery, offering unparalleled outcomes for patients undergoing tissue reconstruction following congenital anomalies, trauma, or tumor resection. The success rate of complex tissue reconstruction using free flaps has consistently increased over the last 30 years, according to several studies currently exceeding 95 percent [1,2,3,4,5,6,7]. However, despite advancements in surgical techniques and perioperative care, free tissue transfer is not without the risk of several complications, including microvascular and peri-anastomotic thrombosis, leading to flap failure, necrosis, and subsequent patient morbidity. Prompt intervention is essential to salvage tissue viability and prevent adverse outcomes in such cases [8,9]. Literature suggests that the critical window for successful flap salvage is within 48 h after surgery [10]. Fibrinolytic agents have emerged as vital adjuncts in the arsenal of reconstructive surgeons for managing compromised flaps after free-flap surgery [11,12]. These cause fibrin clots to dissolve, thereby restoring the blood flow to the ischemic tissue. Among the various fibrinolytic agents, streptokinase, urokinase, acylated plasminogen, streptokinase activator complex, and tissue plasminogen activator (tPA) have attracted considerable attention due to their efficacy and safety profiles in clinical practice [11,13]. Streptokinase, a bacteria-derived fibrinolytic agent, functions by forming a complex with plasminogen and converting it into plasmin, the active enzyme responsible for fibrinolysis. Its wide-ranging activity makes it suitable for managing compromised flaps, particularly in cases of extensive thrombosis. Urokinase acts directly on plasminogens to generate plasmin, facilitating clot dissolution. Its rapid onset of action and minimal systemic side effects make it the preferred choice for managing acute flap ischemia [5]. Acylated plasminogen, a modified form of native plasminogen, offers enhanced stability and specificity for fibrinolytic activity. Its synthetic nature allows for precise dosing and targeted fibrinolysis, making it a promising option for flap salvage procedures. The streptokinase activator complex, a recombinant protein engineered to mimic the action of streptokinase, presents a safer alternative with reduced immunogenicity and improved pharmacokinetic properties. Tissue plasminogen activator (tPA), a crucial regulator of endogenous fibrinolysis, has shown remarkable efficacy in restoring blood flow to compromised flaps. Its selective targeting of fibrin-rich thrombi minimizes systemic bleeding complications, making it an attractive option for salvage procedures in free-flap surgery. The development of recombinant tPA variants has further refined its clinical utility, offering improved stability and enhanced fibrin specificity [14,15]. While fibrinolytic agents hold significant promise in managing compromised flaps, their clinical use necessitates careful consideration of patient-specific factors, such as the extent of ischemia, underlying comorbidities, and risk of bleeding complications. Additionally, optimal dosing regimens and administration protocols are areas of ongoing research that maximize efficacy while minimizing adverse effects.

In this systematic review, we aim to elucidate the current landscape of fibrinolytic agents in the clinical management of the salvage of compromised free flaps.

## 2. Methods

This systematic review was registered in PROSPERO, the International Prospective Register of Systematic Reviews (protocol number = CRD42024503255). This study was conducted according to the guidelines on Preferred Reporting Items for Systematic Reviews and Meta-Analyses (PRISMA) [16].

### Search Strategy and Article Selection

A systematic literature search was performed using the online databases PubMed and Web of Science. The following search term strategy was used.

(“flap*” OR “tissue transfer” OR “microsurg*” OR “microvasc*”) AND (“streptokinase” OR “fibrin*” OR “urokinase” OR “rt-PA” OR “tissue plasminogen activator” OR “thromboly*”) AND (“salvage” OR “rescue”)

Additionally, the MeSH database on PubMed was screened using the following MeSH terms.

(“Surgical Flaps” [Mesh] OR “Myocutaneous Flap” [Mesh] OR “Free Tissue Flaps” [Mesh] OR “Perforator Flap” [Mesh]) AND (“Urokinase-Type Plasminogen Activator” [Mesh] OR “Fibrinolytic Agents” [Mesh] OR “Thrombolytic Therapy” [Mesh])

The literature search was performed in April 2024, with no restrictions concerning the publication year. The articles were then independently screened by three authors (P.M., R.W., and M.M.). Inclusion criteria were case studies, retrospective and prospective studies, interventional clinical studies, case-control studies, and randomized controlled trials regarding the clinical use of fibrinolytic agents for flap salvage in humans in English or German. Exclusion criteria were reviews, editorials and opinion pieces, letters to the editor, study protocols, non-human studies, and cadaver studies. The authors (P.M. and M.M.) independently gathered all search results in an Excel file (Microsoft Excel 2016 32-bit, Redmond, WA, USA) and screened the literature according to the determined inclusion and exclusion criteria. Afterward, they compared their data, and in case of any discrepancies, they reconciled with a third reviewer (A.H.). Information regarding the year of publication, authorship, number of cases, type of flaps, type of thrombosis, fibrinolytic agent used, method of application, timing of revision, flap survival, and adverse events was also collected. The articles were then subsequently categorized based on their level of evidence according to the 2011 American Society of Plastic Surgeons (ASPS) (Arlington Heights, IL, USA) Evidence Rating Scales [17].

## 3. Results

### 3.1. Literature Search

**Figure 1:**
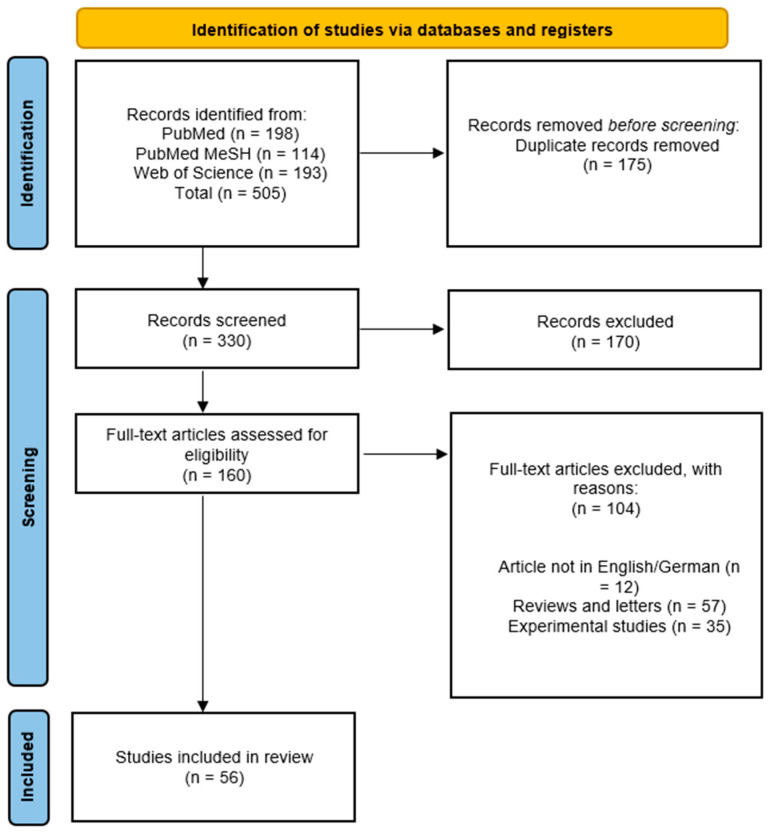
PRISMA Flow Diagram. PRISMA: Preferred Reporting Items for Systematic Reviews and Meta-Analyses.

The literature on PubMed and Web of Science search resulted in a total of 505 studies. Cross-referencing screening did not yield any supplementary articles in accordance with our search criteria and methodology. After the removal of duplicates, 330 records were included for screening. The titles, abstracts, and, if available, full-text articles were reviewed, resulting in the exclusion of 170 studies that did not align with the study question. The remaining 160 studies were screened using the stated inclusion and exclusion criteria. Twelve studies were excluded for not being in English or German, 57 studies were excluded for being reviews and letters, and 35 studies were excluded for being experimental studies. This resulted in 56 studies covering the clinical use of fibrinolytic agents for flap salvage (Figure 1). A brief overview is demonstrated in Table 1.

### 3.2. Study Characteristics

The 56 studies included in the analysis were published from 1987 to 2023. The majority of the studies found were case reports (n = 25), followed by retrospective cohort studies (n = 21) and case series (n = 10), totaling 459 flaps treated with fibrinolytic agents for flap salvage following microvascular compromise. No randomized controlled trials or prospective studies were found.

### 3.3. Evidence Level

The 56 articles that met the inclusion criteria were classified based on their level of evidence. 25 studies were classified as level V, 10 as level IV, and 21 as level III, with 0 studies each for levels II and I.

### 3.4. Flap Survival

Of the 459 flaps treated with fibrinolytic agents, data on flap survival were missing for 27 flaps. Of the 432 patients with described outcomes, 272 (63%) were salvaged and 160 (37%) were lost.

### 3.5. Types of Flaps

All flaps were free flaps used for defect coverage of various sizes. Out of all 459 flaps, 285 (62.1%) were not further described regarding the type of flap. Among the described flaps, there were 26 (5.7%) adipocutaneous flaps, 57 (12.4%) fasciocutaneous flaps, 64 (13.9%) musculocutaneous flaps, 14 (3.1%) muscle flaps, 11 (2.4%) osteocutaneous flaps, and 2 (0.4%) bone flaps.

### 3.6. Type of Thrombosis

Among all 459 cases, information about the type of thrombosis was missing for 216. Of the 243 flaps with the described thrombosis types, 69 (28.4%) had arterial thrombosis, 146 (60.1%) had venous thrombosis, and 28 (11.5%) had both arterial and venous thrombosis.

### 3.7. Method of Application

In 238 (52%) cases, the method of administration was documented, and in 221 (48%) cases, no information was available about the method of application of the fibrinolytic agent, or the information was not assignable. The most common method of application was direct administration to the arterial pedicle, with 215 documented cases (90.3%). In 14 cases (5.9%), the fibrinolytic agent was administered subcutaneously or into the arterial pedicle. Specifically, in 4 cases (1.7%), the fibrinolytic agent was administered subcutaneously; in another 4 cases (1.7%), catheter-directed thrombolysis over 24 h was performed; and in 1 case (0.4%), the fibrinolytic agent was administered intravenously. Of the 215 cases in which the fibrinolytic agent was administered directly into the arterial pedicle, in 47 cases (21.9%), the surgeons allowed systemic circulation of the fibrinolytic agent; in 157 cases (73%), it was prevented in 11 cases (5.1%), and no information was provided. To prevent systemic circulation, the vein was either opened or clamped and sometimes both the artery and the vein were clamped.

### 3.8. Fibrinolytic Agents

**Figure 2:**
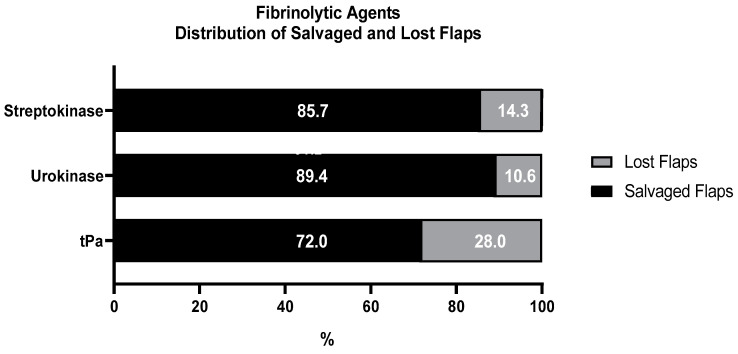
Distribution of salvaged and lost flaps regarding used fibrinolytic. Streptokinase was used in 14, urokinase in 48, and tPa in 118 flap cases. tPa: tissue plasminogen activator.

The most used fibrinolytic agent was tissue plasminogen activator (tPa) [6,13,14,15,18,20,21,24,27,28,29,30,32,33,35,39,42,43,45,47,50,52,53,56,57,58,59,66], with 176 (63.8%) described uses. The second most commonly used fibrinolytic agent was urokinase [5,11,13,24,26,28,29,34,35,38,40,41,46,48,49,51,55,61], with 79 (28.6%) described uses, and the third was streptokinase [27,28,36,37,40,44,54,60,62,63,64,65], with 21 (7.6%) described uses. In 183 cases, the fibrinolytic agent used was not described properly for a quantitative analysis [4,19,22,23,25,31]. The distribution of salvaged and lost flaps concerning the fibrinolytic used, as properly described in the included studies, is illustrated in Figure 2. When tPa was used, the most common dosage ranged between 2 and 10 mg [6,13,14,15,18,20,27,29,32,33,43,45,50,52,53,58,59,66]. Less than 2 mg (1 mg) was only used in one case [6]. More than 10 mg was administered in five cases [24,42,47,57], with the highest dose being 100 mg [42]. In 118 cases, the salvage rate after tPa usage was described, resulting in 85 (72.0%) successfully salvaged flaps and 33 (28.0%) lost flaps. In one study, two patients had to return to the operating theater due to bleeding complications [15], one study described a mild periorbital hematoma [47], another study monitored a small hematoma at the donor side [52], and in one study, the patient required 10 packed red blood cell transfusions (pRBCt) [57]. Three studies [18,33,45] stated that four flaps could only partially be saved. 12 Small skin and/or fat necrosis were seen in five studies [14,20,29,32,58]. Three medium-to large-sized fat necroses were found in two studies (35,41). Senchenkov et al. [6] also mention three fat necrosis. Another patient suffered from fibrous nonunion of a fibula flap that required surgical debridement 1 year after tPa admission [35]. When urokinase was used, the most common dosage ranged between 50.000 and 250.000 units [11,13,24,26,29,34,38,40,41,46,48,49,51,61]. In two cases, dosages of 300.000 [34] and 400.000 [51] were used. In 47 cases, the salvage rate after urokinase usage was described, resulting in 42 (89.4%) salvaged flaps and 5 (10.6%) lost flaps (Figure 2). Regarding bleeding complications, there were two cases with hematoma at the donor side [5], and in the other two cases, there was a need for one packed red blood cell transfusion (pRBCt) each [29]. Three partial necrosis were described in two studies [5,11]. Serletti et al. [26] listed two marginal losses of tissue, one fat necrosis and one fistula, as adverse events. There were also two extensive muscle necrosis [38] and a 50% flap loss described [61].

The streptokinase dosages mostly ranged between 20.000 and 125.000 units [36,40,54,60,62,64,65], with a dosage as low as 7500 only used in one case [44]. In 14 cases, the salvage rate after streptokinase usage was described, resulting in 12 (85.7%) salvaged flaps and 2 (14.3%) lost flaps (Figure 2). Regarding bleeding complications, there was a flap hematoma in one study [40] and one small hematoma on the donor side in another study [63]. Two studies described a flap that was only partially salvaged (20% lost) [44,60], and another two studies described partial flap necrosis in two flaps [36,37]. Lastly, Noordanus et al. [65] described a small, self-healing wound dehiscence. In 253 cases, it was not possible to distinguish between the fibrinolytic agents used and the respective outcomes, or there was no exact description of the fibrinolytic agent. In these 253 cases, 133 (53%) flaps were salvaged, and 120 (47%) flaps were lost.

In some studies, only the complications were described, but it was not possible to distinguish between the fibrinolytic agents used, or the fibrinolytic agent was simply not mentioned. To these complications count two flaps with necrosis [13], 3 partial skin losses [4], and in another two studies, one only partially saved flap in each [19,25].

## 4. Discussion

Within our systematic review, we were able to identify three main fibrinolytic agents used for free-flap salvage.

The most frequently used agent was tissue plasmin activator (tPA). As a serine protease enzyme, its main function is to convert plasminogen, arising from fibrin within the blood clot, to plasmin. Its pharmacology rests upon the breakdown of fibrin crosslinks through plasmin following the dissolution of intravascular blood clots [67]. The regular dosage of tPA for arterial thrombosis and emboli is 0.1 mg/kg body weight.

tPA was used in 118 cases with a dosage range of 2–10 mg, resulting in 85 salvaged free flaps with a low-to-moderate adverse events profile. The dosage of tPA was mostly lower than suggested in acute myocardial infarction or pulmonary embolism, yet it has to be mentioned that the application was given as a bolus injection intra-arterially into the flap artery, with no or minimal systemic affection of tPA in the majority of reported cases. There was only one case of severe hemorrhage and the postoperative need for pRBCt. This single case was within a severe burn victim with the use of continuous tPA administration; hence, the indication was not to salvage a free flap but to prevent thromboembolic events due to burn trauma.

Even though urokinase is the most widespread fibrinolytic agent in interventional radiology, it was only the second most used fibrinolytic agent in free-flap salvage [68]. Urokinase directly cleaves unbound plasminogen in the arteriovenous system plasmin and is not dependent on fibrinogen to release fibrin-based plasminogen. With a total dosage range of 50.000 up to 250.000 units (regular initial dosage in thrombotic events: 4400 IU/kg as loading and 4400 IU/kg/h for 24 h as maintenance dose), 47 free flaps were treated with urokinase, of which 42 survived. Even though the half-life is about 15 min, in several cases within our review, major bleeding was reported; some of them needed pRBCt, indicating a systemic effect. Due to its short half-life, studies of pulmonary embolism and myocardial infarction suggest that there is a potential risk of re-thrombosis within 30 min after successful thrombolysis [69]. To reduce the risk of re-thrombosis, yet to profit from the short half-life, the use of heparin post-interventionally is strongly suggested with close monitoring of aPTT [70].

Finally, streptokinase was used to salvage free flaps in 14 cases, with 12 being successful. 20.000–125.000 units were administered for free-flap salvage (regular dose for arterial thrombosis: 250.000 IU as loading and 100.000 IU as maintenance over 6 h). Streptokinase indirectly uses the free plasminogen to be converted into plasmin, thereby initiating the lysis of blood clots within the vascular system. Due to the dual plasmin-activation, first, the fibrin bound and second, the unbound plasminogen, there are two half-lives. For fibrin-bound plasmin, it is about 11–13 min, and for unbound plasmin, it is about 80 min [68,70,71,72].

Within the streptokinase group, only small hematomas were recognized as adverse events from the application of the agent. One of the major advances seems to be the dual half-life of streptokinase, which might lead to reduced use of postoperative heparin and further reduce the risk of re-thrombosis compared with urokinase.

The quality of available data regarding the level of evidence is quite low. Most studies are either case reports or case series. Retrospective studies are typically large single-center flap analyses that also examine fibrinolysis, but they often do not focus on it explicitly. The topic of fibrinolysis, or flap salvage procedures, in general, is usually mentioned only briefly, with inadequate descriptions of adverse events, administration protocols, and dosages. Most crucially, data quality is insufficient to establish a clear link between interventions and possible outcomes, which makes it impractical to conduct a meta-analysis.

## 5. Conclusions

Although the occurrence of vascular compromise seems to be a rare event, it is crucial to act rapidly to successfully salvage the free flaps. In our systematic review, fibrinolytic agents such as tPA, urokinase, and streptokinase were used to salvage free flaps after the development of vascular compromise due to thrombosis.

These agents have proven to be safe and effective as salvage procedures when administered intra-arterially into the flap artery, with venous drainage branching off the regular vascular system.

Still, it has to be mentioned that most of the investigated literature consists of case reports and some case series, limiting the validity of this review. The retrospective nature of the literature with inconsistent documentation allows only a basic statement.

There seems to be a lack of prospective controlled studies. Future scientific endeavors should consider the flap type, time of occurrence of vascular compromise, which fibrinolytic agent was used, how it was administered, and how the dosage was determined. Finally, a statement should be presented if the salvage was successful or failed to salvage the flap.

## Figures and Tables

**Table 1:** Overview of reviewed articles.

Author	Level of Evidence	Cases	Fibrinolytic Agent	Method of Application	Timing of Revision p.o.	Flap Survival	Adverse Events
Kim et al., 2023 [5]	3	16	Urokinase mean 69,688 U (range: 30,000–100,000 U)	Infused into the arterial pedicle with venous drainage—no systemic circulation	45.4 h (range: 24–88 h)	13/16 (81.3%)	Two flaps with transient partial necrosis, 2 cases with hematoma at the donor site
Coriddi et al., 2022 [18]	3	26	tPa mean 4.4 ± 2.5 mg	Arterial injection: When possible, a vein was opened—systemic circulation	58.9 h ± 51.5 h	16/26 (55.4%)	One flap was only partially salvaged, and adverse events n/a
Chang et al., 2011 [13]	3	33	7× urokinase mean 75,000 U; 26× tPa mean 2.5 mg	n/a	n/a	28/33 (84.8%)	Two flaps with fat necrosis
Rinker et al., 2007 [15]	3	15	tPa 2.5 mg, a second dose of 2.5 mg in 8 flaps	Arterial injection, vene is clamped—no systemic circulation	14.1 h ± 13.9 h	10/15 (66.7%)	Two patients went back to the OR due to bleeding complication
Chang et al., 2016 [19]	3	56	n/a	n/a	n/a	28/56 (50%)	One flap was only partially salvaged, and adverse events n/a
Casey et al., 2007 [20]	3	11	tPa 2 mg	Arterial injection, venous anastomosis open to drain—no systemic circulation	n/a	10/11 (90.9%)	One small fat necrosis
Namgoong et al., 2018 [11]	3	6	Urokinase 100,000 U	Arterial injection, vene clamped/opened—no systemic circulation	mean of 50.8 h	6/6 (100%)	1× partial necrosis
Khansa et al., 2013 [21]	3	12	tPa units n/a	n/a	7× intraoperative, 5× n/a	6/12 (50%)	n/a
Mirzabeigi et al., 2012 [22]	3	36	10× tPa, 10× urokinase, 3× streptokinase, 10× n/a—units n/a	Arterial injection—no systemic circulation	n/a	21/36 (64%)	n/a
Panchapakesan et al., 2003 [4]	3	20	n/a	Arterial injection, vene opened—no systemic circulation	mean of 32.8 h in salvaged flaps; mean of 63.8 h in lost flaps	6/20 (30%)	3× partial skin loss
Bui et al., 2007 [23]	3	20	Streptokinase 50,000–250,000 U or tPa 5 to 20 mg	Arterial injection, artery, and vene clamped- no systemic circulation	n/a	13/20	n/a also, regarding partially salvaged
Yii et al., 2001 [24]	3	8	7× urokinase mean 100,000 U; 1× tPa 15 mg	Arterial injection—systemic circulation n/a	n/a	6/8 (0.75%)	n/a
Largo et al., 2018 [25]	3	11	n/a	n/a	n/a	1/11 (9%)	One only partially salvaged, adverse events n/a
Serletti et al., 1998 [26]	3	5	5× urokinase 250,000	Arterial injection—systemic circulation	mean of 3.6 days p.o (1 to 6 days)	5/5 (100%)	2× marginal loss of tissue, 1× fat necrosis, 1× fistula, no other adverse events
Senchenkov et al., 2015 [6]	3	14	tPa—1 × 1 mg, 5 × 2 mg (redone 1x), 2 × 6 mg, 1× 10 mg, 1× unknown	Subcutaneously or arterial injection—systemic circulation n/a	n/a	12/14 (86%)	3× fat necrosis
Tall et al., 2015 [27]	3	6	1× streptokinase, 5× tPa (3–10 mg)	Arterial injection, vene was clamped—no systemic circulation	n/a	5/6 (83%)	n/a
Nelson et al., 2015 [28]	3	16	5× tPa, 8× urokinase, 3× streptokinase	n/a	n/a	n/a	n/a
Nelson et al., 2012 [29]	3	7	5× urokinase 250,000 U, 2× tPa 2 mg	intra-arterial over 30 min—systemic circulation	n/a	6/7 (86%)	1× urokinase (partial salvage—Fat necrosis, transfuse 1 unit pRBCx), 1× pTa fat necrosis, 1× Urokinase Flap loss, transfuse 1 unit pRBC
Bishop et al., 2023 [30]	3	11	tPa	n/a	n/a	n/a	n/a
Selber et al., 2012 [31]	3	63	n/a	n/a	n/a	25/63 (41%)	n/a
Vijan et al., 2007 [32]	3	18	rt-PA between 4 and 10 mg	Intra-arterial or intravenous—systemic circulation n/a	mean of 127 min in salvaged flaps; mean of 192 min in lost flaps	10/18 (56%)	6× superficial skin necrosis, 2× minor fat necrosis (>2 cm), 2× major fat necrosis (<2 cm)
Ihler et al., 2013 [33]	4	3	tPa 2 mg	Multiple injections subcutaneously	mean of 96 h	3/3 (100%)	2× flap only partially salvaged
Anavekar et al., 2011 [34]	4	2	1× urokinase 100,000 U, 1× Urokinase 200,000 U, and another 100,000 the next day	Arterial injection—systemic circulation	1× 21st p.o. day, 1× 30th p.o. day	2/2 (100%)	none
Trussler et al., 2008 [35]	4	2	1× urokinase 1 mL/min for 24 h, Alteplase and Retevase for 24 h (no dosage)	Catheter-directed thrombolysis over 24 h—systemic circulation	1× 12th p.o. day, 1× 6th p.o. day	2/2 (100%)	1× stroke 3 years after thrombolysis (urokinase), 1× fibrous nonunion of fibula flap—required fibro-osseous debridement 1 year after the initial operation (tPa)
Egozi et al., 2011 [36]	4	2	2× streptokinase 20,000–50,000 U	1× infused into the arterial pedicle with venous drainage—no systemic circulation, 1× unknown	1× intraoperative (saved), 1× n/a	1/2 (50%)	1× Partial distal necrosis
Weinzweig et al., 1995 [37]	4	1	Streptokinase	Infused into the arterial pedicle with venous drainage—no systemic circulation	8 h	1/1 (100%)	Partial flap necrosis
Lyons et al., 2005 [38]	4	2	2× urokinase 250,000 U	intra-arterial, clamped—no systemic circulation	within 24 h	2/2 (100%)	2× extensive muscle necrosis
Tamplen et al., 2013 [39]	4	1	1× tPa	intra-arterial—systemic circulation	8th p.o. day	1/1 (100%)	none
Goldberg et al., 1989 [40]	4	6	Streptokinase (2× 125,000 U in 1 flap, 3× 50,000), Urokinase 1× 100,000 U, 1× 50,000 U	6× Arterial injection—5× no systemic circulation, 1× n/a	2× intraoperative, 1 × 3 h, 1 × 7 h, 1 × 9 h (lost), 1 × 16 h p.o.	5/6 (86%)	1× Flap hematoma
D’Arpa et al., 2005 [41]	4	2	1× urokinase 100,000 U, 2× Urokinase 50,000 into other flap	2× Infused into the arterial pedicle with vene opened—no systemic circulation	1× 2nd p.o. day, 1× intraoperative	2/2 (100%)	none
Bonde et al., 2004 [42]	4	2	50 mg rt-Pa (actilyse) and after 15 min another 50 mg	1st dose into the arterial pedicle with vene opened—no systemic circulation/second dose after 15 min systemic	1 × 14 h p.o., 1× 18 h. p.o.	2/2 (100%)	none
Tessler et al., 2014 [43]	5	2	1× tPa 6 mg, 1× tPa 10 mg	closed-loop circuit—no systemic circulation	1× 2nd p.o. day (lost), 1× 5th p.o. day	1/2 (50%)	none
Schubert et al., 1987 [44]	5	1	Streptokinase 7500 U	Arterial and venous branches clamped—no systemic circulation	intraoperative	1/1 (100%)	1× flap partially salvaged (20% lost)
Atiyeh et al., 1999 [45]	5	1	tPa 4 mg	Arterial and venous infusion—systemic circulation	18 h	1/1 (100%)	1× flap partially salvaged (50% lost)
Lee et al., 2013 [46]	5	1	1× urokinase 100,000 U	Arterial injection, flushed out of venae comittans—no systemic circulation	intraoperative	1/1 (100%)	none
Zhang et al., 2021 [47]	5	1	2× tPa 10 mg in one flap = 20 mg	Arterial injection, both veins and artery clamped—no systemic circulation	intraoperative	1/1 (100%)	Mild right periorbital hematoma
Hong et al., 2020 [48]	5	1	Urokinase 55,000 U	Arterial injection, vene ligated—no systemic circulation	144 h	1/1 (100%)	n/a
Barhoum et al., 2020 [14]	5	1	rt-PA 4 mg	Arterial injection, venous anastomosis opened—no systemic circulation	11 h	1/1 (100%)	slight superficial necrosis at the epidermis and a dehiscence at the cranial part
Renaud et al., 1996 [49]	5	1	Urokinase 75,000 U	Arterial injection—systemic circulation n/a	intraoperative	1/1 (100%)	none
Malhotra et al., 2020 [50]	5	1	1× rt-PA 2 mg	Arterial injection, vene opened—no systemic circulation	20 h	1/1 (100%)	none
Hsu et al., 2017 [51]	5	1	1× urokinase 60,000 U	Arterial injection, vene opened, and artery clamped- no systemic circulation	32 h	1/1 (100%)	none
Fudem et al., 1989 [52]	5	1	tPa 4 mg	Intravenous—systemic circulation	4th p.o. day	1/1 (100%)	small hematoma on the donor site
Wimbauer et al., 2022 [53]	5	1	tPa 2 mg	Arterial injection, Artery, and vene clamped—no systemic circulation	12 h	1/1 (100%)	none
Lipton et al., 1987 [54]	5	1	1× streptokinase 60,000 U	Infused into the arterial pedicle with venous drainage—no systemic circulation	41.5 h	1/1 (100%)	none
Agostini et al., 2012 [55]	5	1	1× urokinase 400,000 U	Arterial injection—systemic circulation n/a	12 h	1/1 (100%)	n/a
Chan et al., 2010 [56]	5	1	tPa—dose n/a	Arterial injection—no systemic circulation	5th p.o. day	1/1 (100%)	none
Coeugniet et al., 2019 [57]	5	1	tPa, 1 mg/h = 24 mg	Arterial with Katheter—1 mg/h for 24 h—systemic circulation	day of operation	1/1 (100%)	Required 10 packed red blood cell transfusions.
Ayhan et al., 2009 [58]	5	1	rt-PA 2 mg	Subcutaneously	after 36 h	1/1 (100%)	Small lateral skin and fat necrosis
Tran et al., 2006 [59]	5	1	1× rt-PA 4 mg after 12 h, 1× rt-PA 2 mg rt-PA, 2 mg on p.o. day 6	intra-arterial—systemic circulation	after 12 h	1/1 (100%)	5 × 5 cm fat necrosis
Tse et al., 2003 [60]	5	1	1× streptokinase 30,000 U	intra-arterial, clamped—no systemic circulation	11th p.o. day	1/1 (100%)	Partial flaps loss 20%
Handschin et al., 2010 [61]	5	1	1× urokinase 100,000 U and another n/a dosis of urokinase on p.o. day 15	Intra-arterial—systemic circulation	3rd p.o. day	1/1 (100%)	50% flap loss
Wechselberger et al., 1998 [62]	5	1	1× streptokinase 100,000 U	Infused into the arterial pedicle with vene opened—no systemic circulation	46 h	1/1 (100%)	none
Cangé et al., 1987 [63]	5	1	Streptokinase 5000 U per hour	Catheter-directed thrombolysis for 41 h—systemic circulation	after 12 h	0/1 (0%)	small hematoma on the donor site
Tonks et al., 1995 [64]	5	1	1× streptokinase 20,000 U	intra-arterial, artery clamped, vene remained open—systemic circulation	intraoperative	1/1 (100%)	none
Noordanus et al., 1993 [65]	5	1	1× streptokinase 100,000 U	Infused into the arterial pedicle with vene opened—no systemic circulation	19th p.o. days	1/1 (100%)	small wound dehiscence—self-healing
Nikkhah et al., 2015 [66]	5	1	rt-PA 4 mg	Infused into the arterial pedicle with vene opened—no systemic circulation	4th p.o. day	1/1 (100%)	n/a

## Data Availability

Due to copyright reasons, the data are not available.

## References

[1] Copelli C., Tewfik K., Cassano L., Pederneschi N., Catanzaro S., Manfuso A., Cocchi R. (2017). Management of free flap failure in head and neck surgery. Acta Otorhinolaryngol. Ital..

[2] Bengur F.B., Saadoun R., Moroni E.A.M., Khan N.I., Bottegal M.T.B., Sridharan S., Kubik M.W., Solari M.G. (2023). Venous Thromboembolism Rates After Free Flap Reconstruction of the Head and Neck Region. Ann. Plast. Surg..

[3] Modarressi A., Schettini A.V., Rüegg E.M., Pittet-Cuénod B. (2018). Venous thromboembolism events after breast reconstructions with DIEP free flaps in 192 consecutive case. Ann. Chir. Plast. Esthet..

[4] Panchapakesan V., Addison P., Beausang E., Lipa J.E., Gilbert R.W., Neligan P.C. (2003). Role of thrombolysis in free-flap salvage. J. Reconstr. Microsurg..

[5] Kim J.H., Yoon S., Kwon H., Oh D.Y., Jun Y.J., Moon S.H. (2023). Safe and effective thrombolysis in free flap salvage: Intra-arterial urokinase infusion. PLoS ONE.

[6] Senchenkov A., Lemaine V., Tran N.V. (2015). Management of perioperative microvascular thrombotic complications—The use of multiagent anticoagulation algorithm in 395 consecutive free flaps. J. Plast. Reconstr. Aesthetic Surg..

[7] Escandón J.M., Ciudad P., Mayer H.F., Pencek M., Mantilla-Rivas E., Mohammad A., Langstein H.N., Manrique O.J. (2023). Free flap transfer with supermicrosurgical technique for soft tissue reconstruction: A systematic review and meta-analysis. Microsurgery.

[8] Askari M., Fisher C., Weniger F.G., Bidic S., Lee W.P.A. (2006). Anticoagulation therapy in microsurgery: A review. J. Hand Surg..

[9] Smit J.M., Acosta R., Zeebregts C.J., Liss A.G., Anniko M., Hartman E.H.M. (2007). Early reintervention of compromised free flaps improves success rate. Microsurgery.

[10] Shen A.Y., Lonie S., Lim K., Farthing H., Hunter-Smith D.J., Rozen W.M. (2021). Free Flap Monitoring, Salvage, and Failure Timing: A Systematic Review. J. Reconstr. Microsurg..

[11] Namgoong S., Yang J.P., Jeong S.H., Han S.K., Kim W.K., Dhong E.S. (2018). Pharmacological thrombolysis: The last choice for salvaging free flaps. J. Plast. Surg. Hand Surg..

[12] Brouwers K., Kruit A.S., Hummelink S., Ulrich D.J.O. (2020). Management of free flap salvage using thrombolytic drugs: A systematic review. J. Plast. Reconstr. Aesthetic Surg..

[13] Chang E.I., Mehrara B.J., Festekjian J.H., Da Lio A.L., Crisera C.A. (2011). Vascular complications and microvascular free flap salvage: The role of thrombolytic agents. Microsurgery.

[14] Barhoum F., Tschaikowsky K., Koch M., Kapsreiter M., Sievert M., Müller S., Goncalves M., Traxdorf M., Scherl C. (2020). Successful free flap salvage surgery with off-label use of Alteplase: A case report, review of the literature and our free flap salvage algorithm. Int. J. Surg. Case Rep..

[15] Rinker B.D., Stewart D.H., Pu L.L.Q., Vasconez H.C. (2007). Role of recombinant tissue plasminogen activator in free flap salvage. J. Reconstr. Microsurg..

[16] Moher D., Liberati A., Tetzlaff J., Altman D.G. (2009). Preferred Reporting Items for Systematic Reviews and Meta-Analyses: The PRISMA Statement. J. Clin. Epidemiol..

[17] (2011). American Society of Plastic Surgeons ASPS Evidence Rating Scales. www.plasticsurgery.org/Documents/medical-professionals/health-policy/evidence-practice/ASPS-Rating-Scale-March-2011.pdf.

[18] Coriddi M., Myers P., Mehrara B., Nelson J., Cordeiro P.G., Disa J., Matros E., Dayan J., Allen R., McCarthy C. (2022). Management of postoperative microvascular compromise and ischemia reperfusion injury in breast reconstruction using autologous tissue transfer: Retrospective review of 2103 flaps. Microsurgery.

[19] Chang E.I., Zhang H., Liu J., Yu P., Skoracki R.J., Hanasono M.M. (2016). Analysis of risk factors for flap loss and salvage in free flap head and neck reconstruction. Head Neck.

[20] Casey W.J., Craft R.O., Rebecca A.M., Smith A.A., Yoon S. (2007). Intra-arterial tissue plasminogen activator: An effective adjunct following microsurgical venous thrombosis. Ann. Plast. Surg..

[21] Khansa I., Chao A.H., Taghizadeh M., Nagel T., Wang D., Tiwari P. (2013). A systematic approach to emergent breast free flap takeback: Clinical outcomes, algorithm, and review of the literature. Microsurgery.

[22] Mirzabeigi M.N., Wang T., Kovach S.J., Taylor J.A., Serletti J.M., Wu L.C. (2012). Free flap take-back following postoperative microvascular compromise: Predicting salvage versus failure. Plast. Reconstr. Surg..

[23] Bui D.T., Cordeiro P.G., Hu Q.Y., Disa J.J., Pusic A., Mehrara B.J. (2007). Free flap reexploration: Indications, treatment, and outcomes in 1193 free flaps. Plast Reconstr Surg..

[24] Yii N.W., Evans G.R.D., Miller M.J., Reece G.P., Langstein H., Chang D., Kroll S.S., Wang B., Robb G.L. (2001). Thrombolytic Therapy: What Is Its Role in Free Flap Salvage?. Ann. Plast. Surg..

[25] Largo R.D., Selber J.C., Garvey P.B., Chang E.I., Hanasono M.M., Yu P., Butler C.E., Baumann D.P. (2018). Outcome Analysis of Free Flap Salvage in Outpatients Presenting with Microvascular Compromise. Plast. Reconstr. Surg..

[26] Serletti J.M., Moran S.L., Orlando G.S., O’Connor T., Herrera H.R. (1998). Urokinase protocol for free-flap salvage following prolonged venous thrombosis. Plast. Reconstr. Surg..

[27] Tall J., Björklund T.C., Skogh A.C.D., Arnander C., Halle M. (2015). Vascular complications after radiotherapy in head and neck free flap reconstruction: Clinical outcome related to vascular biology. Annals of Plastic Surgery.

[28] Nelson J.A., Fischer J.P., Grover R., Kovach S.J., Low D.W., Kanchwala S.K., Levin L.S., Serletti J.M., Wu L.C. (2015). Vein grafting your way out of trouble: Examining the utility and efficacy of vein grafts in microsurgery. J. Plast. Reconstr. Aesthetic Surg..

[29] Nelson J.A., Kim E.M., Eftakhari K., Low D.W., Kovach S.J., Wu L.C., Serletti J.M. (2012). Late venous thrombosis in free flap breast reconstruction: Strategies for salvage after this real entity. Plast. Reconstr. Surg..

[30] Bishop J.L., Vasudev M., Garcia N., Heslop G., Pham T.T., Hicks M.D., Chowdhury F., Grayson J.W., Goddard J.A., Tjoa T. (2023). Effect of Perioperative Antithrombotics on Head and Neck Microvascular Free Flap Survival After Anastomotic Revision. Otolaryngol.—Head Neck Surg..

[31] Selber J.C., Soto-Miranda M.A., Liu J., Robb G. (2012). The survival curve: Factors impacting the outcome of free flap take-backs. Plast. Reconstr. Surg..

[32] Vijan S.S., Tran N.V. (2007). Microvascular breast reconstruction pedicle thrombosis: How long can we wait?. Microsurgery.

[33] Ihler F., Matthias C., Canis M. (2013). Free flap salvage with subcutaneous injection of tissue plasminogen activator in head and neck patients. Microsurgery.

[34] Anavekar N.S., Lim E., Johnston A., Findlay M., Hunter-Smith D.J. (2011). Minimally invasive late free flap salvage: Indications, efficacy and implications for reconstructive microsurgeons. J. Plast. Reconstr. Aesthetic Surg..

[35] Trussler A.P., Watson J.P., Crisera C.A. (2008). Late free-flap salvage with catheter-directed thrombolysis. Microsurgery.

[36] Egozi D., Fodor L., Ullmann Y. (2011). Salvage of compromised free flaps in trauma cases with combined modalities. Microsurgery.

[37] Weinzweig N., Gonzalez M. (1995). Free tissue failure is not an all-or-none phenomenon. Plast. Reconstr. Surg..

[38] Lyons A.J., James R., Collyer J. (2005). Free vascularised iliac crest graft: An audit of 26 consecutive cases. Br. J. Oral Maxillofac. Surg..

[39] Tamplen M., Blackwell K., Jahan R., Nabili V. (2013). Salvage of free-flaps in vessel-depleted mandibular osteoradionecrosis cases using catheter-directed thrombolysis and angioplasty. J. Reconstr. Microsurg..

[40] Goldberg J.A., Pederson W.C., Barwick W.J. (1989). Salvage of free tissue transfers using thrombolytic agents. J. Reconstr. Microsurg..

[41] D’Arpa S., Cordova A., Moschella F. (2005). Pharmacological thrombolysis: One more weapon for free-flap salvage. Microsurgery.

[42] Bonde C.T., Heslet L., Jansen E., Elberg J.J. (2004). Salvage of free flaps after venous thrombosis: Case report. Microsurgery.

[43] Tessler O., Vorstenbosch J., Jones D., Lalonde S., Zadeh T. (2014). Heparin-induced thrombocytopenia and thrombosis as an under-diagnosed cause of flap failure in heparin-naive patients: A case report and systematic review of the literature. Microsurgery.

[44] Schubert W., Hunter D.W., Guzman-Stein G., Ahrenholz D.H., Solem L.D., Dressel T.D., Cunningham B.L. (1987). Use of streptokinase for the salvage of a free flap: Case report and review of the use of thrombolytic therapy. Microsurgery.

[45] Atiyeh B.S., Fuleihan N.S., Musharafieh R.S. (1999). Pharmacologic partial salvage of a failing free flap with recombinant tissue plasminogen activator (rt-PA). J. Reconstr. Microsurg..

[46] Lee D.S., Jung SIl Kim D.W., Dhong E.S. (2013). Anterograde intra-arterial urokinase injection for salvaging fibular free flap. Arch. Plast. Surg..

[47] Zhang S.L., Ng H.W. (2021). Primary thrombolysis for free flap surgery in head and neck reconstruction: A case report and review. Arch. Plast. Surg..

[48] Hong S.H., Lee K.T., Pyon J.K. (2020). Salvage of late flap compromise in deep inferior epigastric perforator flaps: To revise or not to revise. Arch. Plast. Surg..

[49] Renaud F., Succo E., Alessi M.C., Legre R., Juhan-Vague I. (1996). Iloprost and salvage of a free flap. Br. J. Plast. Surg..

[50] Malhotra G., Patil R., Komma V.N.R. (2022). Use of Tissue Plasminogen Activator to Salvage a Precious Free Tissue Transfer. J. Hand Microsurg..

[51] Hsu S.Y., Cheng H.T., Manrique O., Hsu Y.C. (2017). Anterograde injection of low-dose urokinase salvages free anterolateral thigh flap. Medicine.

[52] Fudem G.M., Walton R.L. (1989). Microvascular thrombolysis to salvage a free flap using human recombinant tissue plasminogen activator. J. Reconstr. Microsurg..

[53] Wimbauer J.M., Heinrich K.M., Schwaiger K., Pumberger P., Koeninger F., Wechselberger G., Russe E. (2022). Anterograde Injection of Alteplase Salvages Deep Inferior Epigastric Perforator Flap in Reconstructive Breast Surgery. Plast. Reconstr. Surg. Glob. Open.

[54] Lipton H.A., Jupiter J.B. (1987). Streptokinase salvage of a free-tissue transfer: Case report and review of the literature. Plast. Reconstr. Surg..

[55] Agostini T., Lazzeri D., Agostini V., Spinelli G., Shokrollahi K. (2012). Delayed free flap salvage after venous thrombosis. J. Craniofacial Surg..

[56] Chan R.K., Mathy J.A., Przylecki W., Guo L., Caterson S.A. (2010). CASE REPORT Superior Gluteal Artery Perforator Flap Breast Reconstruction Salvage Following Late Venous Congestion after Discharge. Eplasty.

[57] Coeugniet E., Al Yafi M.N., Lafrance D., Danino M.A., Soulez G., Nguyen Q., Harris P. (2019). Microcirculatory Free Flap Failure with Patent Anastomosis Salvaged by in Situ Thrombolysis in Vulnerable Phase Burn. J. Burn. Care Res..

[58] Ayhan S., Uygur S., Kucukoduk I., Sencan A. (2009). Salvage of a congested DIEAP flap with subcutaneous recombinant tissue plasminogen activator treatment. J. Plast. Reconstr. Aesthetic Surg..

[59] Tran N.V., Bishop A.T., Convery P.A., Yu A.Y. (2006). Venous congestive flap salvage with subcutaneous rtPA. Microsurgery.

[60] Tse R., Ross D., Gan B.S. (2003). Late salvage of a free TRAM flap. Br. J. Plast. Surg..

[61] Handschin A.E., Guggenheim M., Calcagni M., Künzi W., Giovanoli P. (2010). Factor v leiden mutation and thrombotic occlusion of microsurgical anastomosis after free TRAM flap. Clin. Appl. Thromb./Hemost..

[62] Wechselberger G., Schoeller T., Ohler K., Anderl H., Ninkovic M. (1998). Flap salvage in a “flow-through” flap by manual thrombectomy plus thrombolytic therapy abstract. J. Reconstr. Microsurg..

[63] Cangé S., Laberge L.C., Rivard G.E., Garel L. (1987). Streptokinase in the management of limb arterial thrombosis following free-flap surgery. Plast. Reconstr. Surg..

[64] Tonks A.M., Rees M. (1995). Streptokinase salvage of a rectus abdominis free flap. Plast. Reconstr. Surg..

[65] Noordanus R.P., Joris Hage J. (1993). Late salvage of a “free flap” phalloplasty: A case report. Microsurgery.

[66] Nikkhah D., Green B., Sapountzis S., Gilleard O., Sidhu A., Blackburn A. (2016). Resurrection of an ALT flap with recombinant tissue plasminogen activator and heparin. Eur. J. Plast. Surg..

[67] Collen D. (1987). Molecular mechanism of action of newer thrombolytic agents. J. Am. Coll. Cardiol..

[68] Ohman E.M., Harrington R.A., Cannon C.P., Agnelli G., Cairns J.A., Kennedy J.W. (2001). Intravenous thrombolysis in acute myocardial infarction. Chest.

[69] Hepburn-Brown M., Darvall J., Hammerschlag G. (2019). Acute pulmonary embolism: A concise review of diagnosis and management. Intern. Med. J..

[70] Maizel A.S., Bookstein J.J. (1986). Streptokinase, urokinase, and tissue plasminogen activator: Pharmacokinetics, relative advantages, and methods for maximizing rates and consistency of lysis. Cardiovasc. Intervent. Radiol..

[71] Khan I.A., Gowda R.M. (2003). Clinical perspectives and therapeutics of thrombolysis. Int. J. Cardiol..

[72] Goodman Gilman A., Hardman J.G., Limbird L.E., Molinoff P.B., Ruddon R.W., Molinoff P.B., Ruddon R.W. (1996). Goodman and Gilman’s the Pharmacological Basis of Therapeutics.

